# Transcriptomic analyses highlight the likely metabolic consequences of colonization of a cnidarian host by native or non-native *Symbiodinium* species

**DOI:** 10.1242/bio.038281

**Published:** 2019-02-27

**Authors:** Mei-Fang Lin, Shunichi Takahashi, Sylvain Forêt, Simon K. Davy, David J. Miller

**Affiliations:** 1 Molecular and Cell Biology, James Cook University, Townsville, QLD 4811, Australia; 2 ARC Centre of Excellence for Coral Reef Studies, James Cook University, Townsville, QLD 4811, Australia; 3 Evolutionary Neurobiology Unit, Okinawa Institute of Science and Technology Graduate University, Okinawa 904-0495, Japan; 4 Division of Environmental Photobiology, National Institute for Basic Biology, Nishigonaka 38, Myodaiji, Okazaki 444-8585, Japan; 5 Ecology and Evolution, Research School of Biology, Australian National University, Canberra, ACT 0200, Australia; 6 School of Biological Sciences, Victoria University of Wellington, Kelburn Parade, Wellington 6140, New Zealand

**Keywords:** Corallimorpharia, Recolonization, Symbiodinium, Symbiosis

## Abstract

Reef-building corals and some other cnidarians form symbiotic relationships with members of the dinoflagellate family *Symbiodinaceae.* As *Symbiodinaceae* is a highly diverse taxon, the physiological interactions between its members and their hosts are assumed to differ between associations. The presence of different symbiont types is known to affect expression levels of specific host genes, but knowledge of the effects on the transcriptome more broadly remains limited. In the present study, transcriptome profiling was conducted on the tropical corallimorpharian, *Ricordea yuma,* following the establishment of symbiosis with either the ‘homologous’ symbiont *Symbiodinium goreaui* (also known as *Cladocopium goreaui*; ITS2 type C1) or ‘heterologous’ symbionts (predominantly *S. trenchii*, which is also known as *Durusdinium trenchii*; ITS2 type D1a) isolated from a different corallimorpharian host (*Rhodactis indosinensis*). Transcriptomic analyses showed that genes encoding host glycogen biosynthesis pathway components are more highly induced during colonization by the homologous symbiont than by the heterologous symbiont. Similar patterns were also observed for several other genes thought to facilitate symbiotic nutrient exchange, including those involved in lipid translocation/storage and metabolite transport. The gene expression results presented here imply that colonization by homologous or heterologous *Symbiodinium* types may have very different metabolic consequences for the *Ricordea* host, supporting the notion that even though some cnidarians may be able to form novel symbioses after bleaching, the metabolic performance of these may be compromised.

This article has an associated First Person interview with the first author of the paper.

## Abstract (precis)

**Summary:** Colonization by the homologous symbiont, *Symbiodinium goreaui,* resulted in greater glycogen synthesis and ammonium assimilation capacity in the host than when it was colonized by a heterologous symbiont (*S. trenchii*).

## INTRODUCTION

Many cnidarians including corals, sea anemones and jellyfish host endosymbiotic dinoflagellates of the family *Symbiodinaceae* (Davy et al., 2012). Translocation of photosynthetic products, including carbohydrates and lipids, from the symbionts supports host respiration, growth and, in the case of stony corals calcification. In return, the host provides inorganic carbon, nitrogen and phosphorus to the symbionts (Davy et al., 2012). The cnidarian-*Symbiodinium* symbiosis is a cornerstone of biologically-enriched coral reef ecosystems.

Whilst some species inherit *Symbiodinium* maternally (vertical transmission), the majority of coral species acquire their symbionts from the environment (horizontal transmission) during the early stages of each generation (Baird et al., 2009). *Symbiodinium* cells isolated from cnidarian hosts can often infect a range of other host species, at least under experimental conditions (e.g. Coffroth et al., 2010; Rädecker et al., 2018; Voolstra et al., 2009; Weis et al., 2001; Yuyama and Higuchi, 2014). Although many aspects of the interaction remain unclear, the establishment of a stable cnidarian-dinoflagellate relationship is thought to involve a complex series of processes including recognition, suppression of the normal host phagocytotic pathway and ultimately metabolite trafficking (Davy et al., 2012; Matthews et al., 2017; Mohamed et al., 2016), however less is known about the molecular events beyond that point.

*Symbiodinium* is a highly diverse genus; nine clades are currently recognized and each of these includes many different phylotypes or species (reviewed by LaJeunesse, 2017; Pochon and Gates, 2010). Physiological characteristics – such as photosynthetic activity, growth and stress tolerance – differ among *Symbiodinium* taxa (Baird and Maynard, 2008; Díaz-Almeyda et al., 2017; Klueter et al., 2015). Initial uptake of potential symbionts appears to be a relatively promiscuous process (Biquand et al., 2017; Cumbo et al., 2013), but there are likely to be several subsequent opportunities for the host to reject phylotypes of *Symbiodinium* that do not fit its physiological requirements (or vice versa; Dunn and Weis, 2009). Each host organism harbors one or several different *Symbiodinium* phylotypes in a polyp or colony, and the dominant *Symbiodinium* phylotype can differ among cnidarian species, seasons, and environmental light and temperature conditions (Baker, 2003; Kuguru et al., 2008; Wilkinson et al., 2015). It is conceivable that the host cnidarian changes the dominant *Symbiodinium* phylotype to adapt to environmental change (Buddemeier and Fautin, 1993; Rouzé et al., 2017).

Symbionts provide carbon and nitrogen metabolites to the host, but the efficiency with which this occurs differs among *Symbiodinium* phylotypes (Baker et al., 2013). The coral *Isopora palifera* naturally associates with either *Symbiodinium* C or D types and nanoscale secondary ion mass spectrometry (NanoSIMS) has clearly demonstrated that the type C *Symbiodinium* fixes and transfers more carbon and nitrogen to its host than does the type D symbiont (Pernice et al., 2015). This work, together with studies conducted in the Davy laboratory (Matthews et al., 2017) implies that genes associated with carbon and nitrogen metabolism in the host are likely to differ markedly in expression levels when different *Symbiodinium* phylotypes are present.

Few data are available concerning the effects of different *Symbiodinium* phylotypes on host gene expression. Yuyama et al. (2011) used high-coverage gene expression profiling (HiCEP) to examine the response of aposymbiotic juveniles of *Acropora tenuis* to two different symbiont types but, although 765 genes were differentially expressed between the two groups, only 33 (some of which may be involved in lipid metabolism) could be annotated and validated. Moreover, the presence of different symbiont types has been shown to affect expression levels of specific host genes (Yuyama et al., 2011). In the case of the symbiotic sea anemone *Exaiptasia pallida* (‘*Aiptasia*’), colonization by heterologous symbionts (*S. trenchii*) essentially induced an expression pattern that was intermediate between the symbiotic (i.e. colonized to a similar density by *S. minutum*=ITS2 type B1) and aposymbiotic states with respect to several pathways associated with symbiosis (Matthews et al., 2017).

A number of investigations have established that the growth and/or nutrition of cnidarian hosts may be compromised by the ‘wrong’ symbiont type, and the Matthews et al. (2017) study highlights the transcriptional consequences of colonization by a heterologous symbiont at a fixed time point. However, to our knowledge, the transcriptional effects in the host during the process of colonization by a heterologous symbiont have not yet been investigated.

In the present study, the temporal effects of colonization by heterologous or homologous *Symbiodinium* taxa on the host transcriptome were examined. For this purpose, the tropical corallimorpharian, *Ricordea yuma*, served as host and was infected with either the native (‘homologous’) *Symbiodinium goreaui* (ITS2 type C1) or the non-native (‘heterologous’) *Symbiodinium trenchii* (ITS2 type D1a), an opportunistic, thermally tolerant species, isolated from a different corallimorpharian host (*Rhodactis indosinensis*). Transcriptomic analyses indicate major differences between hosts harboring different *Symbiodinium* species. We discuss the influences of the two symbiont species on host gene expression and consequent implications for host physiology.

## RESULTS AND DISCUSSION

### Uptake of *Symbiodinium* in bleached *Ricordea*

**Fig. 1.**
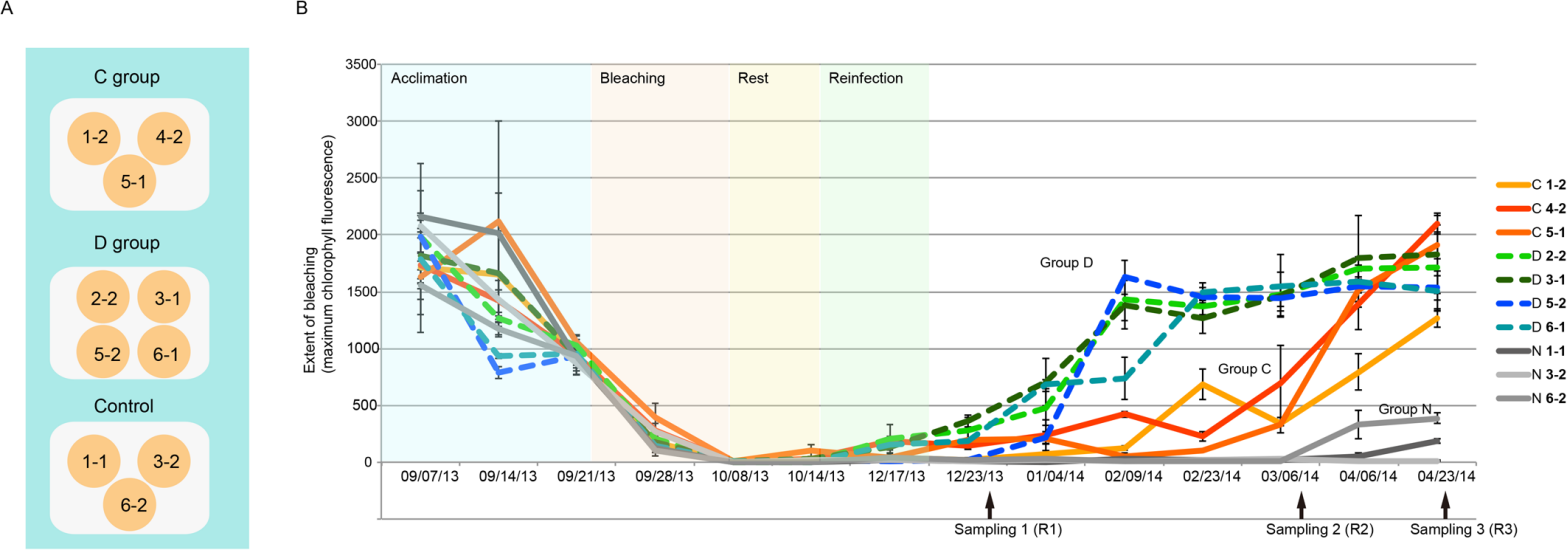
**Experimental design and symbiont density changes during the reinfection study.** (A) Distribution of sample clones in the three experimental tanks. The control group polyps were not experimentally reinfected. Polyps in the C and D treatment groups were inoculated with *Symbiodinium goreaui* or *S. trenchii*, respectively. (B) Symbiont density during the reinfection experiment. The points indicate values of nocturnal maximum quantum yield of photosystem II (Fm) for polyps in the control (N) group (gray solid lines), C group (colored solid lines), and D group (dashed lines) throughout the experiment. The arrows indicate the points at which sampling for RNA extraction was carried out. For detail of sample labelling, refer to Fig. S1.

As described above, bleached *Ricordea* specimens inoculated with homologous (ITS type C1) or heterologous (predominantly ITS2 type D1a) symbionts, and the control (not inoculated) group are henceforth referred to as Groups C, D and N respectively. Following the bleaching treatment, the brownish coloration characteristic of ‘normal’ *Ricordea* was completely absent but gradually recovered in Groups C and D, but not in Group N (Fig. S1). To quantify the bleaching and the recovery, the intensity of chlorophyll *a* fluorescence (*Fm*) emitted from *Symbiodinium* within *Ricordea* tissues was monitored (Fig. 1). Measured *Fm* values were close to zero in all samples after the bleaching treatment but after four months had recovered to initial levels in samples inoculated with *Symbiodinium* (Groups C and D), but not in the non-inoculated control group (Group N; Fig. 1). The recovery was significantly faster in Group D than Group C (approximately 2.8 times faster), suggesting that the infectivity of *S. trenchii* is higher than for *S. goreaui* and/or the growth rate *in hospite* of the former is higher. A major determinant of primary infectivity is cell size, with smaller *Symbiodinium* phylotypes infecting the host faster (Biquand et al., 2017). A small but significant difference (Mann–Whitney *U*-test=−4.63, *P*<0.001) in cell size was observed between the *Symbiodinium* species used in the present study; the size estimate for Clade C1 (*S*. *goreaui*) was 6.45±0.619 µm (*n*=426) and for Clade D (*S. trenchii*) was 6.23±0.692 µm (*n*=418) (Fig. S2). However, both of these values fall into the ‘small’ category for Symbiodiniaceae types and are substantially below the exclusion limit for corals and the sea anemone *Aiptasia* (Biquand et al., 2017). Consequently, symbiont size is unlikely to be a major factor in uptake of symbionts. Although other factors cannot be ruled out (see, for example, Parkinson et al., 2018), the faster recovery of Group D samples is therefore assumed to reflect faster proliferation of *S. trenchii in hospite*.

### Differential gene expression during the re-establishment of symbiosis

As indicated in the methods section, symbiont typing confirmed that the symbiont suspension to which treatment Group D were exposed consisted predominantly (>90%) of *S. trenchii*, but by the end of the experiment (after R3), the predominant symbiont in three members of this treatment group was *S. goreaui*. On this basis, the R3 transcriptomic data for the samples in question were excluded from analyses. As the Group N individuals remained bleached at the R3 time point, it is likely that *S. goreaui* was present at low levels in the heterologous symbiont preparation, ultimately increasing in representation in three of the Group D treatment group members. Searching the raw reads for *S. trenchii* diagnostic ITS2 sequences confirmed the presence of *S. trenchii* in all members of treatment Group D at earlier time points, but this approach cannot provide quantitative data, thus the relative proportions of *S.trenchii* and *S. goreaui* at R1 and R2 are unknown.

**Fig. 2.**
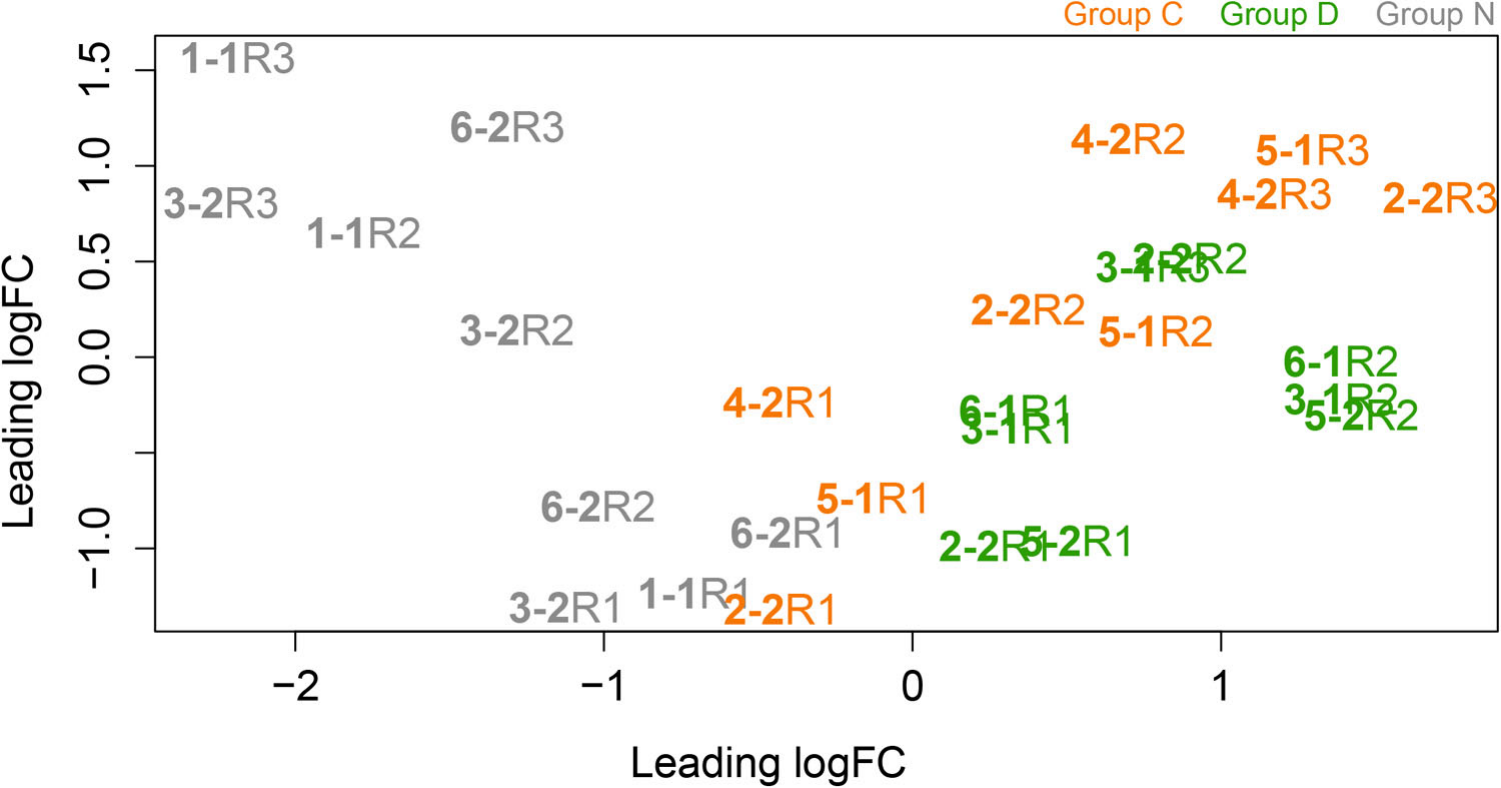
**Relationships between samples based on multidimensional scaling performed using the edgeR package.** Distances on the plot represent the biological coefficient of variation (BCV) between samples. BCV=0.2978. R1, R2 and R3 correspond to the sampling points, sample labeling and grouping as in Fig. 1. Samples in the control groups are marked in gray, samples in C group are in orange and samples in D group are in green.

Nevertheless, to explore the consequences of colonization by heterologous *Symbiodinium* preparations, multidimensional scaling analysis (MDS) was applied to compare the host gene expression patterns between the three treatment groups (Groups C, D and N) during colonization (Fig. 2). At the first colonization time point (R1), all three groups showed similar gene expression patterns, but the patterns differed between the three groups at the later time points (R2 and R3; Fig. 2; Fig. S1), indicating that symbiont identity had a major influence on the expression of many host genes.

**Fig. 3.**
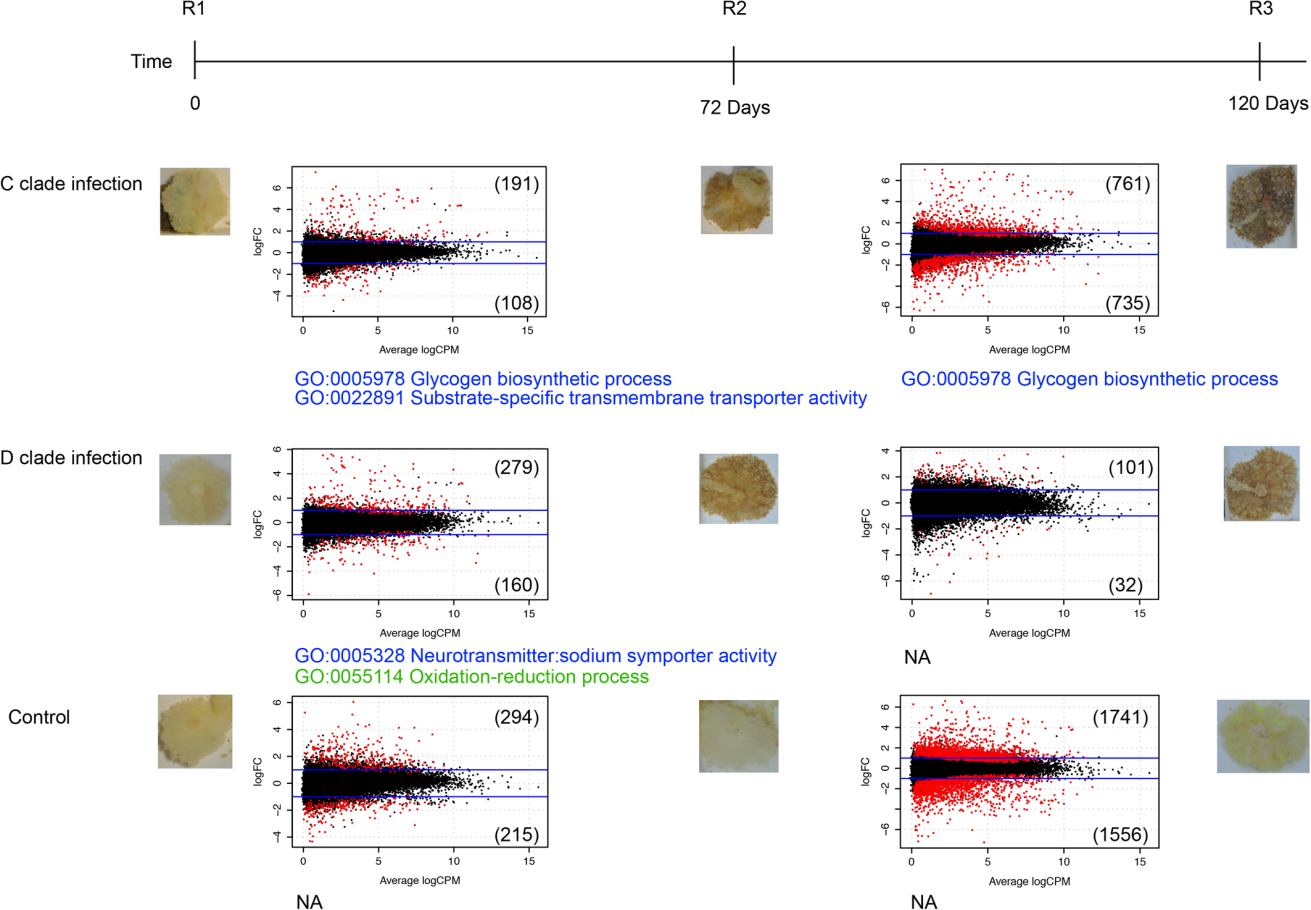
**Dot plots of log-fold-change versus log-cpm (counts per million) at each stage in the three treatment groups, with differentially expressed genes highlighted (5% FDR).** In each case, the blue lines indicate twofold change. In each case, the comparisons were based on sampling point 2 versus sampling point 1, and sampling 3 versus sampling point 1. Numbers of differentially expressed genes are indicated in parentheses above and below the blue lines. Over-represented GO terms are summarized below the plots. Blue, upregulated GO; green, downregulated GO; NA, no significant GO over-representation.

**Fig. 4.**
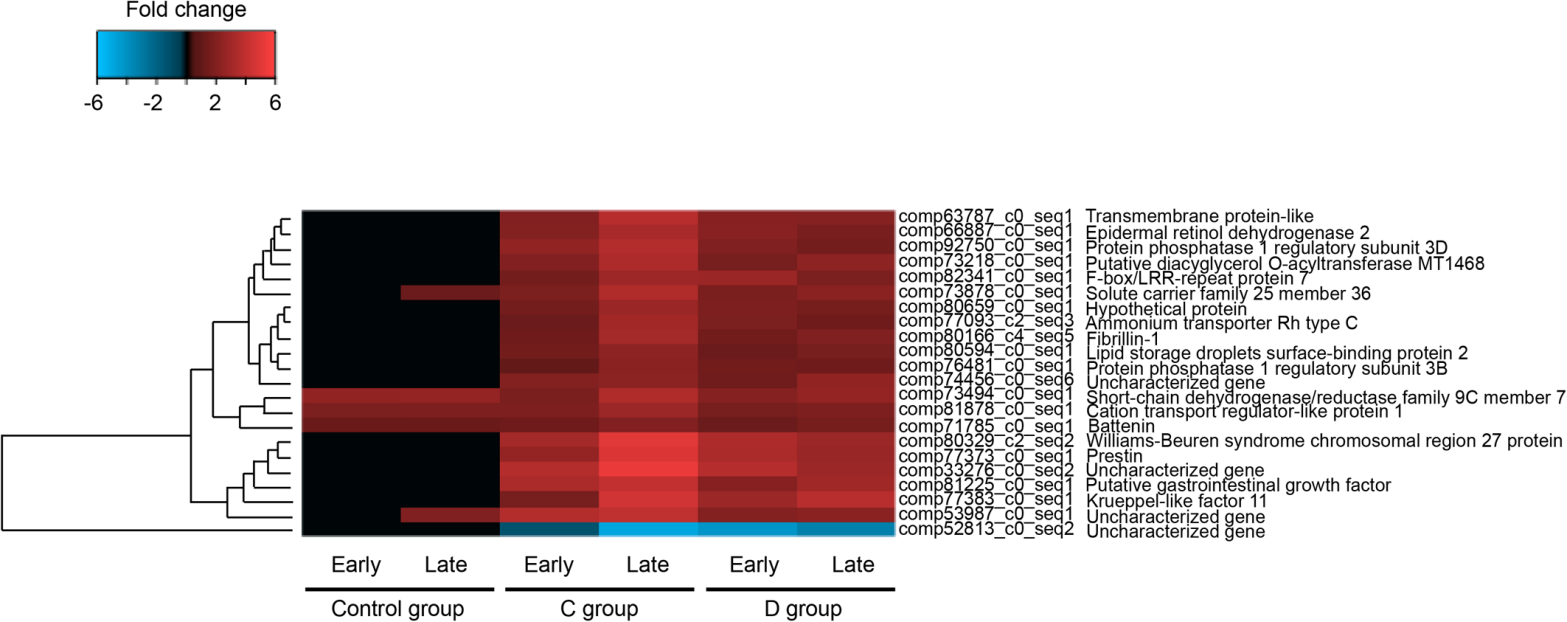
**Genes differentially expressed in the three treatment groups.** Information on the gene families and predicted functions is given in Table S3.

Based on the comparisons of later stages and time zero stages (R1 versus R2 and R1 versus R3), the dispersion patterns of differentially expressed transcripts at each stage in each treatment group are shown as Fig. 3. Using a significance cutoff of FDR<0.05, 22 transcripts were differentially expressed in all three treatment groups (Fig. 4). Of the 22 transcripts, 17 contained open reading frames encoding >100 amino acid residues; 16 of these correspond to annotated proteins and only one cannot be characterized (Table S3). Of these 22 transcripts, 17 were differentially expressed in the C and D treatment groups only (Fig. 4). For most of the genes, differential expression was much more extensive at the late (R3) time point.

### Gene ontology analyses

Gene ontology (GO) analyses indicated that GO terms were significantly over-represented in Group C and/or D samples, but not in Group N samples, during the colonization processes (Fig. 3). The term ‘glycogen biosynthetic process’ (GO:0005978) was over-represented amongst upregulated genes in the R2 and R3 stages of Group C samples, and ‘oxidation-reduction process’ (GO:0055114) was over-represented amongst downregulated genes at the R2 stage in Group D samples. The genes responsible for over-representation of these GO terms were examined further, as described below.

#### Glycogen biosynthesis is enriched in the native Symbiodinium association

**Fig. 5.**
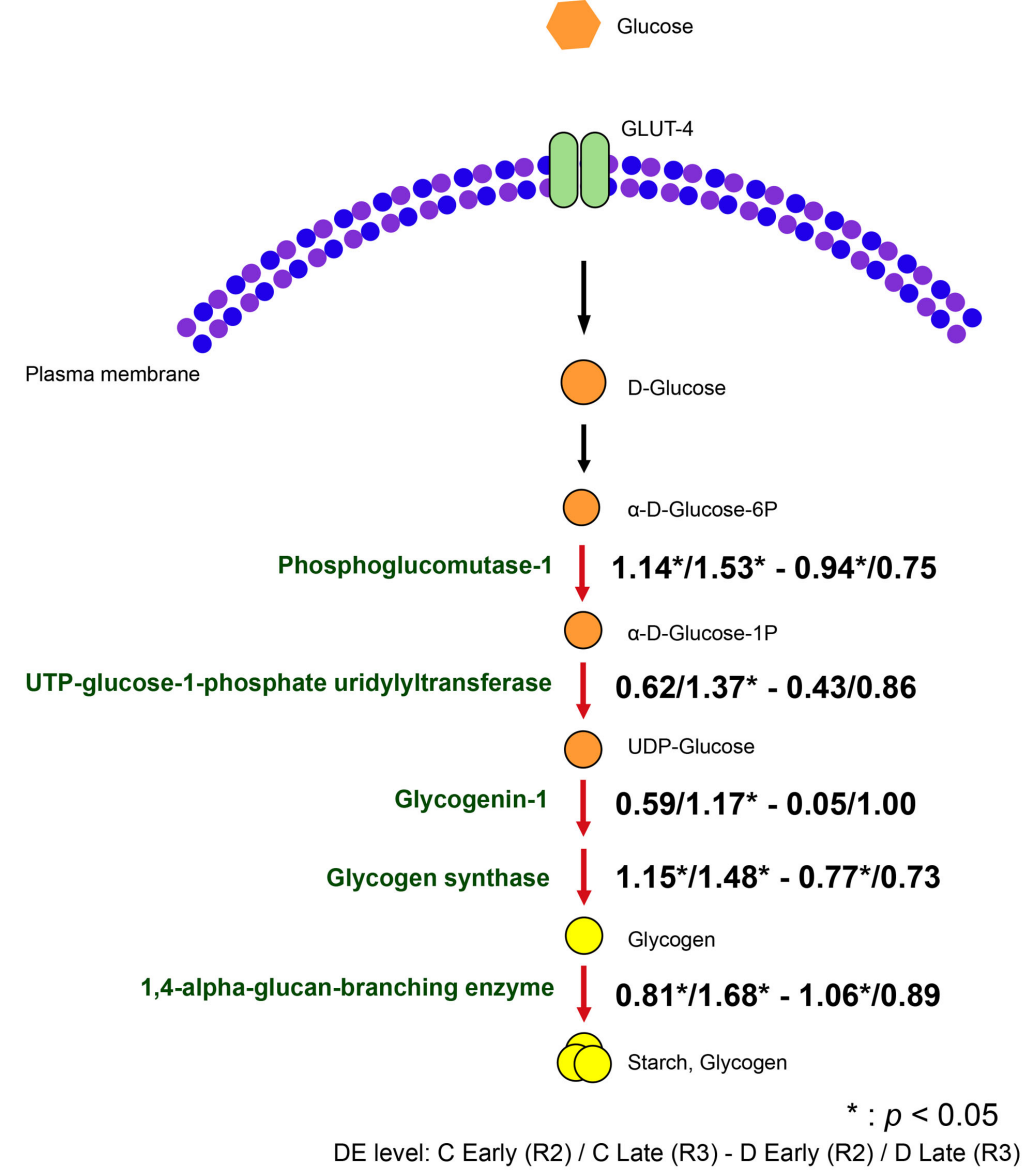
**Differential expression of host genes involved in glycogen biosynthesis during colonization by *Symbiodinium goreaui* (C) or *S. trenchii* (D).** Arrows highlighted in red indicate upregulation of expression relative to levels at the first sampling point. The values represent the logFC of expression at early stage (R2) and late stage (R3) of both groups. (*) indicates the FDR<0.05.

As in other animals, symbiotic cnidarians synthesize glycogen from glucose as a short-term energy storage system (Kopp et al., 2015), and glucose is thought to be the major photosynthetic product transferred from symbiont to host (Burriesci et al., 2012; Hillyer et al., 2017). In the present experiment, three genes involved in glycogen biosynthesis, phosphoglucomutase-1, glycogen synthase and 1,4-alpha-glucan-branching enzyme, were highly upregulated in both colonization stages R2 and R3 in Group C and in the R2 colonization stage of Group D (Fig. 5). Also belonging to the same GO term, UTP-glucose-1-phosphate uridylyltransferase and glycogenin-1 were significantly differentially expressed only in the final colonization stage (R3) of Group C. Although the GO term ‘glycogen biosynthesis’ was not over-represented in the Group D samples, some individual genes in this category were significantly upregulated, including glycogen synthase, phosphoglucomutase-1 and 1,4-alpha-glucan-branching enzyme. However, these genes were not significantly over-represented in the late colonization stage of Group D. Overall, levels of expression of genes involved in glycogen biosynthesis in the D group samples were approximately half of those in C group samples, implying impaired glycogen/starch storage in the D group relative to the C group. Consistent with this result, Matthews et al. (2017) observed lower expression of glycan branching enzyme (the final step in the pathway shown in Fig. 5) and sugar transporters in *Aiptasia* colonized with heterologous *S. trenchii* than with the homologous symbiont (*S. minutum*), while Rädecker et al. (2018) observed the highest gross carbon fixation in anemones colonized with the native *Symbiodinium* phylotype.

In addition to upregulation of genes encoding the catalytic activities involved in glycogen biosynthesis, two regulatory proteins were also upregulated. Protein phosphatase 1 regulatory subunits 3B and 3D (PP1R3B and PP1R3D) both contain carbohydrate-binding domains and act to facilitate glycogen synthesis by suppressing the rate at which PP1 inactivates (dephosphorylates) glycogen phosphorylase and stimulating the rate at which it activates (phosphorylates) this enzyme, effectively driving the reaction in favor of glycogen biosynthesis. Of note, levels of induction of both regulatory proteins were higher in the *S. goreaui*-colonized than in the *S. trenchii*-colonized polyps (Fig. 4).

The upregulation of genes involved in glycogen biosynthesis observed following colonization by *Symbiodinium* is consistent with the translocation of photosynthetically-derived glucose from symbiont to host, and active storage of glucose as glycogen (and starch) in the corallimorpharian host. These data are consistent with reports of higher levels of expression of glycogen-related genes in corals at noon in comparison with those at night (Ruiz-Jones and Palumbi, 2015). Kopp et al. (2015) demonstrated apparent carbon translocation from symbiont to coral host by visualizing external glucose incorporation into glycogen granules in oral epidermal cells, implying that photosynthetically-derived carbon may be stored in coral tissue (e.g. Battey and Patton, 1984; Muscatine et al., 1984; Patton and Burris, 1983; Patton et al., 1977). In a variety of cnidarian-*Symbiodinium* associations, glucose is a major symbiont-derived mobile product (Burriesci et al., 2012; Hillyer et al., 2017; Kopp et al., 2015; Streamer et al., 1993; Whitehead and Douglas, 2003) and the observed upregulation of glycogen biosynthesis during recovery from bleaching supports a central metabolic role for glucose in the corallimorpharian-dinoflagellate symbiosis. The lower levels of expression of glycogen biosynthetic enzymes observed in the corallimorpharians colonized by *S. trenchii* rather than *S. goreaui*, and the faster growth of the former species *in hospite*, are consistent with the idea that less glucose is transferred to the host by *S. trenchii* than by *S. goreaui*. This idea is supported by physiological data indicating that *Isopora palifera* and *Aiptasia* colonized by ITS type D *Symbiodinium* rather than the ‘natural’ symbionts have lower rates of carbon translocation (Leal et al., 2015; Pernice et al., 2015). Lower rates of fixed carbon export by *S. trenchii* relative to several other *Symbiodinium* types (Cantin et al., 2009; Matthews et al., 2017) are assumed to explain the fast growth of ITS type D *Symbiodinium* at an early stage of coral symbiosis (Yuyama and Higuchi, 2014), and may also underlie the patterns observed here in *Ricordea*.

#### Symbiosis results in altered expression of genes involved in ammonia assimilation

The GO term oxidation-reduction process (GO:0055114) was over-represented in R2 stage samples from treatment Group D; some of the genes captured under this GO term were also differentially expressed in treatment Group C (Tables 1 and 2). Two genes involved in nitrogen assimilation were downregulated in both the C and D treatment groups: glutamate dehydrogenase (GDH), which is responsible for the incorporation of ammonium into α-ketoglutarate, and delta-1-pyrroline-5-carboxylate dehydrogenase (ALDH4A1), a mitochondrial enzyme responsible for the production of glutamate from glutamate semialdehyde during breakdown of either proline or ornithine. Based on sequence similarity comparisons, the GDH that is differentially expressed in symbiosis is assumed to be the NADP+-specific variant (Catmull et al., 1987), which is restricted to cnidarians amongst the Metazoa. Like other animals, cnidarians also have a (mitochondrial) GDH that uses both NAD+ and NADP+, but these two classes of protein are clearly distinct; based on BlastP comparison, the *Ricordea* sequence in question matches the *Acropora digitifera* NADP+-specific GDH (XP_015776429.1) with an e-value of 0.0 (overall score 823; identity 83% and coverage 92%) whereas the *A. digitifera* mitochondrial type (XP_015752533.1) matching e-value was only 1e-22 (overall score 180; identity 28% and coverage 76%).

**Table 1.**
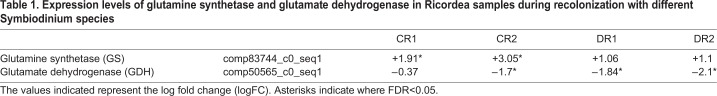
**Expression levels of glutamine synthetase and glutamate dehydrogenase in Ricordea samples during recolonization with different Symbiodinium species**

**Table 2.**
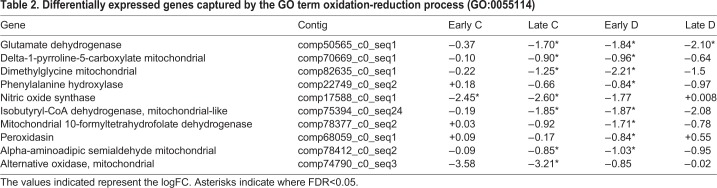
**Differentially expressed genes captured by the GO term oxidation-reduction process (GO:0055114)**

Both GDH and glutamine synthetase (GS) are thought to play important roles in regulating host cytoplasmic ammonium levels in the coral-dinoflagellate symbiosis (Yellowlees et al., 1994). Both of these enzymes are able to function in ammonium assimilation, but do so with very different kinetics – in the case of animal GS proteins, the value of the Michaelis constant (Km) for ammonium is typically around 10 µM, whereas the corresponding figure for GDH is about two orders of magnitude higher; in *Acropora*, the Km of the NADP-specific GDH for ammonium has been estimated as 9.2 mM (Catmull et al., 1987). During the re-establishment of symbiosis with the homologous *S. goreaui*, not only was GDH downregulated, but also GS was significantly upregulated (Table 1). However, GS was apparently not upregulated in the D treatment group. Given the lower Km of GS for ammonium, these results imply that host cytoplasmic ammonium concentrations will be lower in the presence of the homologous symbiont (*S. goreaui*) than in the heterologous situation. One interpretation of the observed activation of host GS in the symbiotic state is that decreasing cytoplasmic ammonium levels impose nutrient limitation on the symbiont, enabling the host to restrict *Symbiodinium* biomass (Davy et al., 2012; Matthews et al., 2017), but another is that the host must maintain low cytoplasmic ammonium levels to prevent collapse of the pH gradient across the symbiosome membrane (Miller and Yellowlees, 1989). Whatever its physiological role, at this stage it is not clear why GS expression is activated by the homologous but not a heterologous symbiont.

The observed upregulation of a homolog of the human Rh type C protein in the symbiotic state has a precedent in the symbiotic sea anemone *Aiptasia* (Lehnert et al., 2014), and may also be relevant to ammonia metabolism in corallimorpharians because the mammalian Rh proteins are ammonia channels. However, the algal Rh proteins are thought to be channels for CO_2_ rather than ammonium (Huang and Peng, 2005), and the cellular location of the cnidarian Rh protein is unknown, therefore its role in symbiosis is unclear.

### Other metabolic aspects of the establishment of symbiosis

The transition from an aposymbiotic to a symbiotic state involves many metabolic adjustments beyond those documented above. Previous observation of well-fed aposymbiotic *Aiptasia* has revealed a metabolic shift from autotrophy to heterotrophy (Oakley et al., 2016). Prior to symbionts being introduced in our study, the corallimorpharians were essentially starved – to induce and maintain the aposymbiotic state, they were maintained in shaded ambient light in filtered seawater and were not fed. Consequently, the upregulation of some genes observed during the course of colonization may simply reflect the downregulation of catabolic processes. Examples of this are dimethylglycine dehydrogenase (DMGDH), phenylalanine hydroxylase (PAH) and isobutyryl-CoA dehydrogenase (ACAD8) (Table 2).

### NGFR and NOS – indicators of coral health?

Nitric oxide (NO) is used as a signaling molecule across the animal kingdom, and diverse roles have been demonstrated in different cnidarians (Colasanti et al., 2010). Nitric oxide synthase (NOS) has been implicated in bleaching (the loss of symbionts) in several cnidarians, but the mechanism is unclear. It has been suggested that, in *Aiptasia*, nitric oxide production essentially serves as an ‘eviction notice’ to symbionts, NO potentially combining with superoxide generated under stress to produce peroxynitrite, which induces cell death and bleaching (Perez and Weis, 2006; but see Hawkins and Davy, 2013). It was initially unclear whether NO generated during bleaching is a product of the host or *Symbiodinium* (Perez and Weis, 2006; Trapido-Rosenthal et al., 2005), though it has more recently been shown that both partners can synthesize NO and that it plays an important signaling role in the bleaching cascade (Hawkins and Davy, 2012; Hawkins et al., 2013). In the present case, a nitric oxide synthase (NOS) that has high similarity to that of *Discosoma striata* (NCBI accession no. AAK61379) was downregulated throughout colonization in the C-treatment group but not in the D-treatment group (Table 2). In *Acropora,* NOS activity has been localized to the endoderm, which is also the location of the symbionts (Safavi-Hemami et al., 2010). During temperature-induced bleaching, elevated levels of NOS in corals and anemones are correlated with the upregulation of host caspase-like enzyme activity (Hawkins et al., 2013, 2014). The downregulation of this gene observed in corallimorpharians colonized by *S. goreaui* suggests that low NOS activity could be an indicator of coral health.

Members of the mammalian tumor necrosis factor (TNF) family were characterized based on their ability to induce apoptosis and in the context of immune responses (Pfeffer, 2003), and tumor necrosis factor receptor (NGFR) has been shown to mediate apoptosis in neural cells (Frade et al., 1996). The coral genome encodes a large number of TNF family members (Quistad et al., 2014), two of which were upregulated in response to heat stress in *Acropora hyacinthus* (Barshis et al., 2013), suggesting involvement in the regulation of apoptosis. Moreover, TNF 2, 6 and caspase-8 involved in apoptosis were suggested to play a role in signaling across the symbiosome and/or oxidative stress pathways in *Aiptasia* when colonized by heterologous symbionts (*S. trenchii*) (Matthews et al., 2017). In the present case, the downregulation of a homolog of NGFR 16 was observed in both reinfection groups, though not at the final sampling point for the *S. trenchii*-colonized animals (data not shown), which could be interpreted in terms of a requirement to suppress cell death processes during symbiosis (Dunn and Weis, 2009).

### Active nutrient allocation

In addition to the implied importance of glucose export and glycogen storage in symbiotic cnidarians, data from our infection study are also consistent with lipid and/or sterol translocation from symbiont to host (Davy et al., 2012; Hillyer et al., 2016, 2017; Kopp et al., 2015; Matthews et al., 2017; Rädecker et al., 2018). Annotated genes found to be upregulated during the establishment of symbiosis included some implicated in lipid/sterol translocation in other symbiotic cnidarians. These include NPC2 (however, in our study this gene was not significantly expressed during the late colonization stage of Group D) (Dani et al., 2014; 2017; Ganot et al., 2011; Kuo et al., 2010; Lehnert et al., 2014; Sproles et al., 2018preprint) and the lipid storage droplet surface-binding protein 2 (Lehnert et al., 2014) (Fig. 4).

Four of these common differentially expressed genes in Groups C and D are membrane-bound proteins, some of which are transport proteins. In addition to the Rh type C (RHCG) protein discussed above, which is involved in transport of ammonium or possibly CO_2_, a Cl^−^/HCO_3_^−^ transport protein known as prestin (solute carrier family 26 member 5, SLC26A5) was upregulated in the symbiotic state. Prestin belongs to the sulfate permease (SulP) protein family, and like several other SLC26 proteins, it contains both sulfate transporter and STAS (Sulphate Transporter and Anti-Sigma factor antagonist) domains. Three SLC26 proteins (SLC26α, β, γ) have previously been identified in the coral *Stylophora pistillata*, but ubiquitous expression of SLC26β suggests that it does not function in symbiosis or calcification (Zoccola et al., 2015). Phylogenetic analyses indicate that prestin has a close relationship with SLC26β (Fig. S3). The fact that expression of this potential HCO_3_^−^ transporter is upregulated during colonization by *Symbiodinium* suggests a role in symbiosis that deserves further exploration.

### Conclusions

Transcriptomic analyses demonstrated that the expression of genes related to the glycogen biosynthetic pathway was higher in hosts containing *S. goreaui* than those containing *S. trenchii*, implying that in the *Ricordea* association *S. goreaui* may translocate more photosynthetically-fixed carbon to the host than *S. trenchii*. The two *Symbiodinium* species also differed with respect to impact on host pathways of ammonium assimilation, with only *S. goreaui* causing upregulation of the high-affinity ammonium assimilation enzyme, glutamine synthase. These results are consistent with previous studies that have shown that symbioses with different *Symbiodinium* taxa are not functionally equivalent (DeSalvo et al., 2010; Loram et al., 2007; Matthews et al., 2017).

A number of previous studies imply the importance of optimal nutritional exchange for optimal performance of cnidarian-*Symbiodinium* associations. For example, although association with an ITS clade D symbiont could increase the thermal tolerance of the *Acropora tenuis* holobiont (Berkelmans and van Oppen, 2006), this was at the ‘cost’ of a negative impact on host growth rate (Little et al., 2004; Jones and Berkelmans, 2010). In the present experiment, several of the *Ricordea* individuals originally infected with *S. trenchii* had largely reverted back to the ‘native’ *S. goreaui* at the final time point (Table S2; note that data for these individuals at that time point were excluded from the transcriptomic analysis), implying optimal performance of the native association and host involvement in symbiont selection (Smith et al., 2017; Stat et al., 2009). However, the mechanism(s) by which cnidarian hosts might select optimal symbionts are unknown.

## MATERIALS AND METHODS

### Sample collection

*Ricordea yuma* polyps originally collected from the Great Barrier Reef (18°25′35.20″S, 146°41′10.91″E) were maintained at Reef HQ aquaria (Townsville, Australia) for several years prior to the start of the work described here. On 23 August 2012, 12 polyps of *Ricordea* were transferred to MARFU at James Cook University and, within 1 h of dispatch, were placed in a 1000 l tank that received a constant flow of seawater (3000 l h^−1^) at an average water temperature of 26.15±0.016°C (as recorded by HOBO Light/Temperature Data Loggers, Onset Corp.). All the samples were exposed to the same shaded ambient light condition. Prior to the experiment, six samples were split to produce six pairs of genetically identical samples. After 2 months, the split samples had fully recovered, resulting in complete polyps with similar diameters (Fig. S1). The remaining six *Ricordea* polyps were used to construct the reference transcriptome (described below).

### *Symbiodinium* isolation, identification and cell size measurement

Prior to the experiment, *Symbiodinium* genotyping was conducted on the corallimorpharians available to us. *Symbiodinium goreaui* (ITS2 type C1) and *S. trenchii* (ITS2 type D1a) were isolated from *R. yuma* and another tropical corallimorpharian, *Rhodactis indosinensis,* respectively. This survey indicated that these two corallimorphs host specific *Symbiodinium* species. Because antibiotic treatment may act as a selective force during *Symbiodinium* culturing (Santos et al., 2001), and it has been suggested that the stress susceptibilities of *Symbiodinium in hospite* differ from those of freshly isolated cells (Bhagooli and Hidaka, 2003), in the present experiment, freshly isolated *Symbiodinium* strains were used for the inoculation. During the isolation process, tissue samples (4–6 tentacles) were ground with a micro sample pestle and 2 ml tube. Once homogenized, the tissue slurry was transferred to a 15 ml Falcon tube, 8 ml 1-micron filtered seawater (FSW) were added, and the sample was vortexed thoroughly and centrifuged at 860× ***g*** for 3 min. The supernatant containing corallimorpharian tissue was discarded. The process was repeated at least four times by resuspension in 8 ml FSW followed by centrifugation (860× ***g*** for 3 min) to pellet the algal symbionts. The algal pellet was re-suspended in 2 ml FSW for later use. Isolated *Symbiodinium* cells were counted with a hemacytometer and diluted to approximately 800 cells ml^−1^ (FSW) before being used for inoculation.

*Ricordea* tissue specimens were cut and preserved in 70% ethanol for genomic DNA extraction following the method of Chen and Yu (2000). For the identification of symbionts present in tissue samples, Polymerase Chain Reaction (PCR) was used to amplify the internal transcribed spacer 2 region (ITS2) with the modified primers ‪ITSintfor2 5′-GAATTGCAGAACTCCGTG-3′ and ITSrev 5′-GGGATCCATATGCTTAAGTTCAGCGGGT-3′ (LaJeunesse and Trench, 2000). Following the supplier's (MyTaq; Bioline, Australia) recommended protocol, samples were denatured for 1 min at 95°C, and then subjected to 30 PCR cycles of 15 s at 95°C, 15 s at 50°C, and 10 s at 72°C. The PCR products were directly sequenced, and then assembled using DNAStar (Lasergene, USA) for *Symbiodinium* typing, which was conducted before the menthol treatment and after the *Symbiodinium* reinfection for all samples.

In order to measure the sizes of the two *Symbiodinium* species used here, measurements of two diameters of a cell intersect at right angles to one another were made for each of more than 400 individual cells, using the CellSens software (Olympus, Japan) in conjunction with an Olympus XB53 microscope.

### Chlorophyll fluorescence measurement

To measure the chlorophyll fluorescence yield of the symbiotic corallimorpharians, maximum chlorophyll fluorescence (*F*_m_) (Maxwell and Johnson, 2000) was measured using a mini PAM fluorometer (Walz, Germany) by applying the saturation pulse in the presence of measuring light. For the measurement of *F*_m_, the mini PAM fiberoptic probe was positioned close (<20 mm) to the corallimorpharian in order to standardize the intensity of the measuring light and saturation pulse. The settings used for each measurement (three measurements per sample per time point) were: suppression of unavoidable background signal (AUTO-ZERO), detection of the photosynthetic active radiation (PAR), saturation pulse intensity (12), electronic signal gain (8), and intensity of measuring light (7).

### Bleaching treatment

To achieve bleaching, samples were incubated in menthol as described in Wang et al. (2012) for 11 days, after which the samples were very pale. The menthol/FSW was prepared by diluting a 20% (w/v) menthol stock with FSW and was used to bleach *Ricordea* at concentration of 0.58 mM. The treatment duration was 8 h, after which samples were allowed to recover for 16 h in fresh FSW, before again changing the FSW. With three replicates of each measurement on the polyp surface (note that *Ricordea* polyps have flat faces), the maximum chlorophyll fluorescence measured by a mini PAM fluorometer (Walz, Germany) was used to indicate the symbiotic state of the samples. Although *Fm*=0 typically indicated a bleached state, very low fluorescence values could still sometimes be detected in apparently bleached specimens (*F*_m_≤15), possibly due the presence of a residual symbiont population at very low density. Samples were used for experiments following maintenance in the absence of menthol for 7 days.

### Inoculation of *Symbiodinium* into the host

Bleached *Ricordea* specimens were separately inoculated with the homologous (*Symbiodinium goreaui*; ITS type C1) or heterologous (predomoinantly *S. trenchii*; ITS2 type D1a), and are henceforth referred to as Groups C and D respectively. As controls, bleached specimens were maintained without *Symbiodinium* inoculation (Group N). From 14 September 2013 to 24 April 2014, all samples were kept at a depth of 12 cm in three aquaria (each with four polyps), with a 300 l h^−1^ flow rate, and consistent temperature of 26°C and ambient light (Fig. 1A). The inoculation process consisted of injecting 1 ml aliquots of the algal suspension (∼800 cells ml^−1^) into the mouth of each polyp at 3–4 day intervals, always at 17:00 h. The water in each tank was changed before the next inoculation, and the water in the control aquarium was changed on the same days as the experimental aquaria. During the course of the experiment, two individuals were lost (one from the control tank and one from Group C) probably because of the weakness of samples after the bleaching stress, thus, at the end of the experiment was complete, a total of 10 samples were available for analysis.

### Reference transcriptome samples

The reference transcriptome was constructed from six different individuals, under a range of treatments, giving rise to nine individual samples, as summarized in Table S1. *Ricordea* has the ability to regenerate new polyps from fragments within two months, and frequently reproduces asexually by marginal budding (Lin et al., 2013). To increase the range of transcriptional states sampled, one polyp (sample V) was bisected, and an individual produced by marginal budding also sampled (sample I). Dark bleached samples were from polyps that had been maintained in the dark for 3 months under the same water flow and temperature conditions as the other treatments (*Fm*=0). After 8–11 days, menthol-treated *Ricordea* became bleached with PSII activity of *Fm*<180.

### Tissue sampling and RNA extraction

Tissues samples (∼300 mg) for RNA extraction were taken from tentacles with sterile scissors at three time points during the colonization process, and colonization stage determined by the value of *Fm* (Hill et al., 2004; Fig. 1B). These stages were: R1, the first sign of colonization; R2, the point at which the symbionts appeared to be evenly distributed in each polyp; and R3, the point at which full colonization was reached (Fig. S1)*.* Tissues samples were immediately snap-frozen in liquid nitrogen and suspended in Trizol for RNA extraction. Total RNA was extracted using the TRI Reagent (Ambion) protocol, which is based on the Chomczynski and Sacchi (1987) method, and then dissolved in RNase-free water. RNA quality and quantity were assessed using a NanoDrop ND-1000 spectrometer and denaturing gel electrophoresis, using standard methods (Sambrook and Russell, 2001). In order to minimize circadian effects on gene expression, sampling was performed at 08:00 h (about 2–2.5 h after sunrise) in all cases (as suggested in Ganot et al., 2011).

### cDNA library development, sequencing and transcriptome assembly

cDNA libraries were generated using the NEBNext Ultra Directional RNA Library Prep Kit for Illumina RNAseq (New England BioLabs, Inc., USA). To generate the reference transcriptome, a total of 769M raw reads were obtained using the Illumina HiSeq2000 sequencing platform. One of the *Ricordea* (symbiotic) libraries was deeply sequenced (a full lane of 100 bp PE reads), the remaining nine samples spread across a further two lanes, and 100 bp single-end (SE) data collected. For the experimental reinfection samples, libraries were sequenced by the use of two lanes (100 bp PE reads). The libngs program (https://github.com/sylvainforet/libngs) was used for quality trimming. After removal of data with quality scores lower than 30 or shorter than 70 bases, a total of 214M reads remained, from which the reference transcriptome was assembled using Trinity (Haas et al., 2013). Symbiont and host sequences were separated and predicted proteins annotated as described in Lin et al. (2017). The *Ricordea* reference transcriptome has 94,579 contigs with a mean size of 1175 bases and N50 of 1679 bases (NCBI BioProject: PRJNA313487).

### Differential expression analysis

Read data from experimental reinfection samples were mapped to the reference transcriptome using the Bowtie v2.1.0 software (Langmead and Salzberg, 2012). Differential gene expression was inferred by mapping counts using the EdgeR package (Robinson et al., 2010) with an expression level cutoff of 5 counts in more than 10 samples and the GLM approach. An MDS plot was used to investigate the relative similarities of the samples (Fig. 2). For gene ontology analyses, GOseq v1.16.1 (Young et al., 2010) was employed, with a differential expression threshold of FDR<0.05.

### Sequence analysis and function prediction

To investigate the potential functions of genes that have no clear homolog in the NR database, PROSCAN (Combet et al., 2000) was used to scan for protein signatures and functional prediction. Conserved domain searching was based on the CDD search tool at the NCBI conserved domain database (Marchler-Bauer et al., 2015). The TargetP 1.1 (Emanuelsson et al., 2000) and TMHMM v. 2.0 (Krogh et al., 2001) servers were used to predict intracellular location and scan for transmembrane domains, respectively.

## Supplementary Material

Supplementary information
