# Supplementary material for: Transcriptomic analyses highlight the likely metabolic consequences of colonization of a cnidarian host by native or non-native Symbiodinium species
Source: Biol Open. 2019 Feb 27;8(3):bio038281. doi: 10.1242/bio.038281 (PMC6451341; doi:10.1242/bio.038281)
Supplement: Supplementary information [file biolopen-8-038281-s1.pdf]

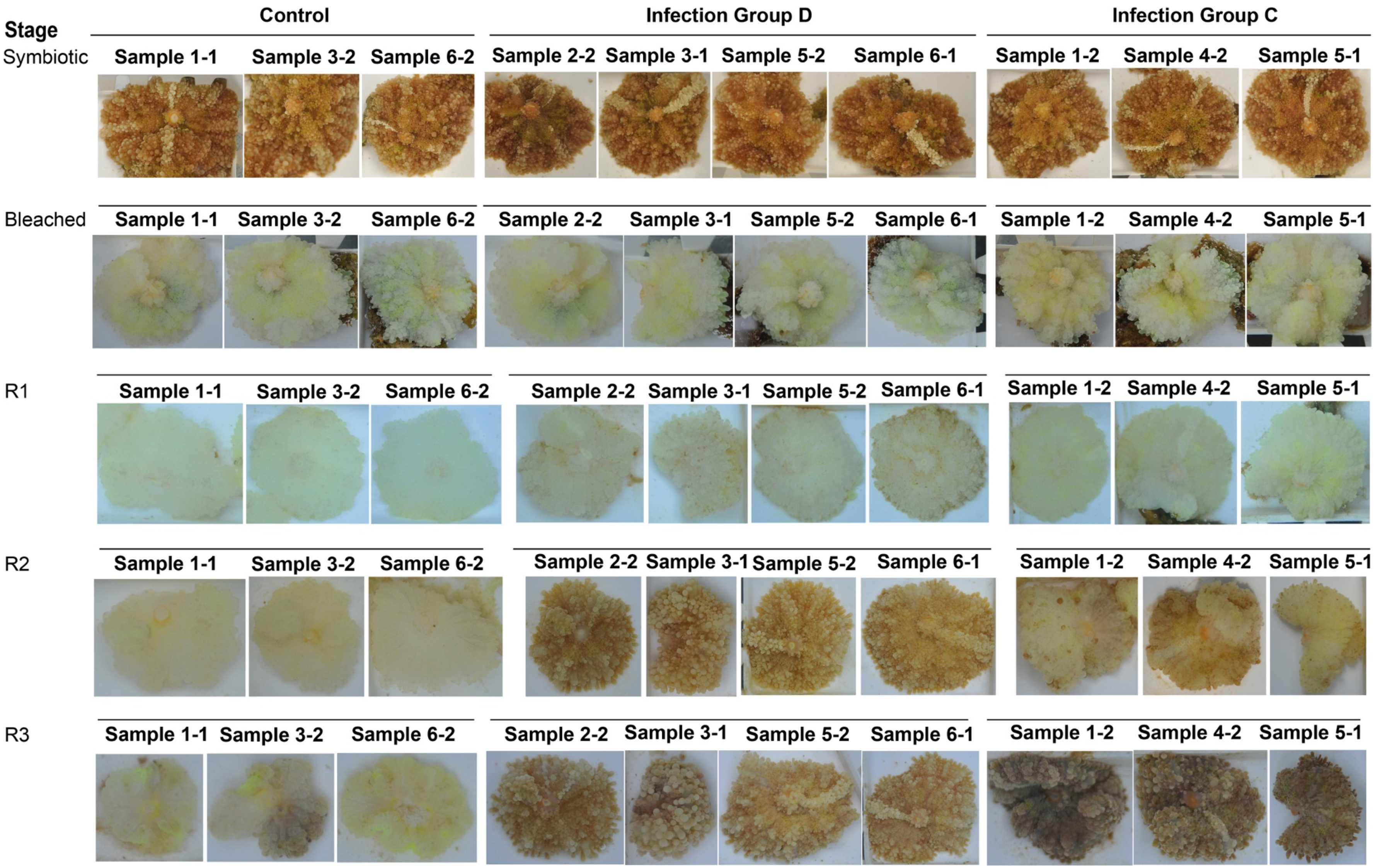

Figure S1. Images of samples in each group throughout the experiment.

Biology Open • Supplementary information

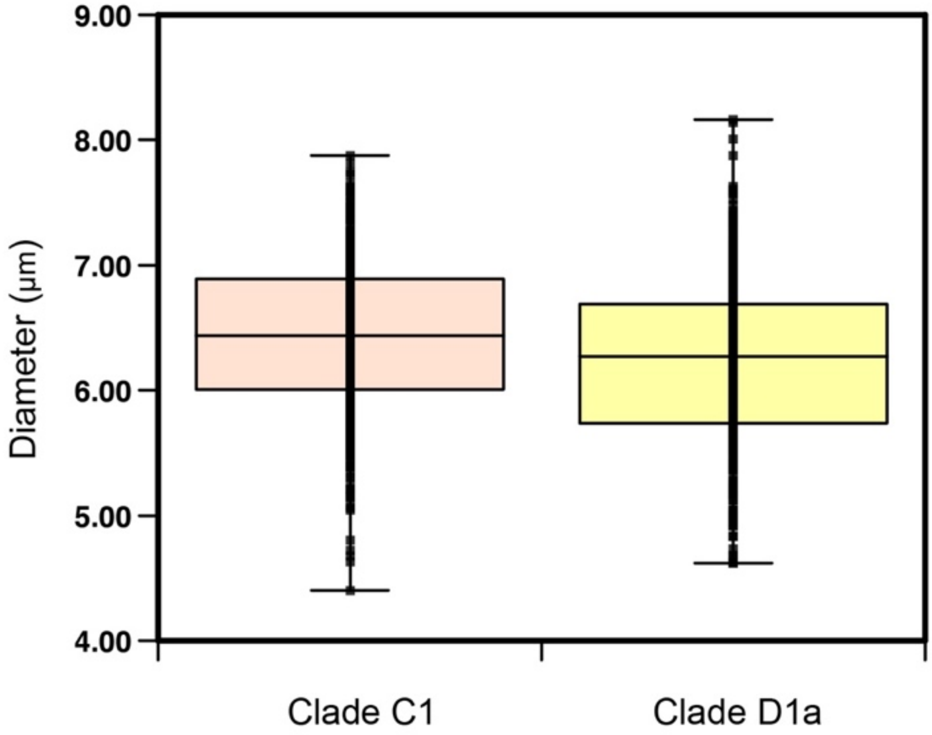

Figure S2. Box plot of cell diameters of the two *Symbiodinium* species included in this study. Number of cells measured for *Symbiodinium goreaui* and *S. trenchii* were 426 and 418, respectively.

Biology Open • Supplementary information

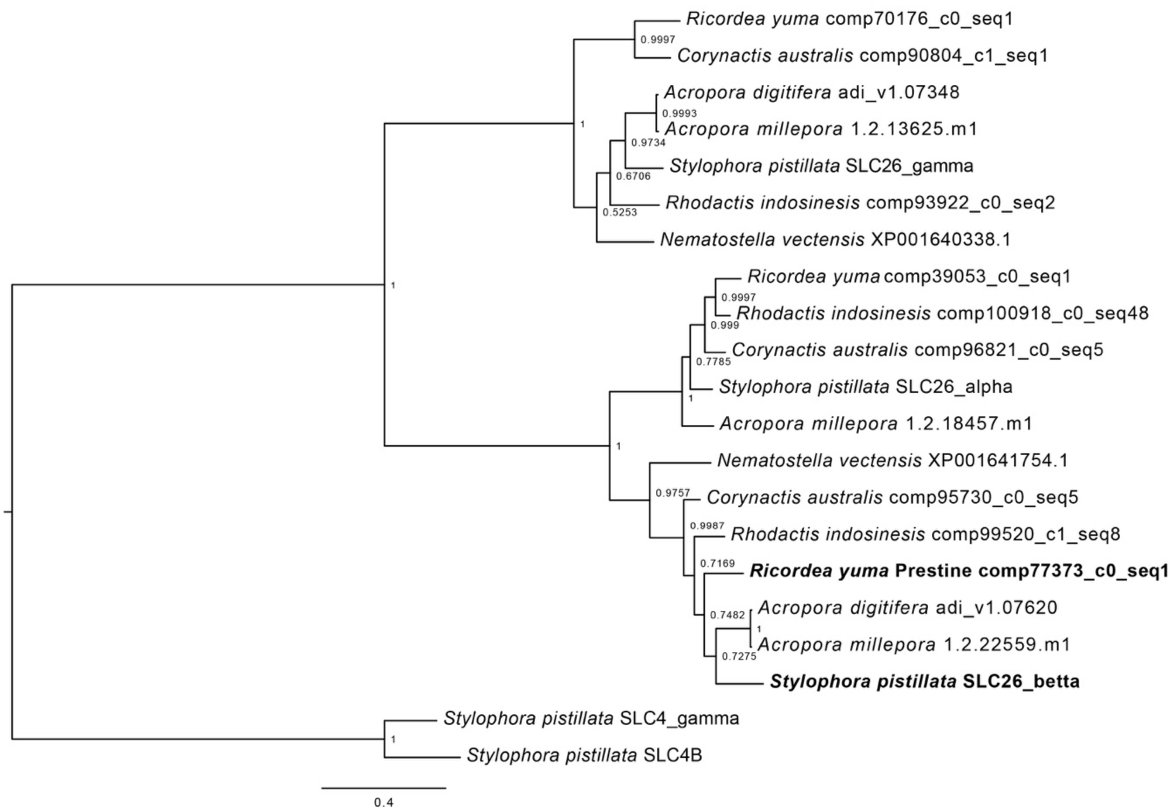

Figure S3. Phylogenetic tree of SLC26 sequences inferred by Bayesian analysis with best-fit model of WAG+G for 2 million generations with first 2000 trees as burnin. The *Ricordea* Pristine (SLC26) and *Stylophora* SLC26β sequences are indicated in bold.

Biology Open • Supplementary information

Table S1. Experimental design for the *Ricordea* reference transcriptome development.

Samples treatment						
Symbiotic	**Sample I**	**Sample II**	**Sample III**		**Sample V1**	
Darkness bleached			Sample IIId	**Sample IVd**		
Menthol bleached	Sample I 1m				Sample V2m	**Sample VIm**

Sample relations:

The samples marked in bold present the mother polyps.

For the daughter polyps, Sample I1m was duplicated from Sample I via asexual reproduction budding, Sample IIId is the same polyp as Sample III but with darkness treatment after sampling for symbiotic condition, and Sample V2m was duplicated from Sample V1 via cutting.

Table S2. A. *Symbiodinium* ITS 2 identification in each sample before and at the final stage of colonization. The annotation of the ITS products after the colonization based on BLASTN against NCBI non-redundant database.

	Group C			Group D				Group control		
Sample	1-2	4-2	5-1	2-2	3-1	5-2	6-1	1-1	3-2	6-2
Before reinfection	Clade C1	Clade C1	Clade C1	Clade C1	Clade C1	Clade C1	Clade C1	Clade C1	Clade C1	Clade C1
After reinfection	Clade C1	Clade C1	Clade C1	Clade C1	CladeD1a	Clade C1	Clade C1	-	-	-
ITS annotation	*Symbiodinium* sp. C1			*Symbiodinium* sp. C1	*Symbiodinium* sp. D1	*Symbiodinium* sp. C1	*Symbiodinium* sp. C	NA	NA	NA
Accession	KU842043			KU842043	MH211594	KU841885	MH236766			
Evalue	9.00E-125			4.00E-118	2.00E-101	3.00E-18	2.00E-96			

Biology Open • Supplementary information

Table S3. Common differentially expressed genes in the infection groups.

Contig	Gene	Domain/Family	TM	SP	Blast Hit	Accession number	Evalue	Predicted function
comp77383_c0_seq1	Krueppel-like factor 11	Zinc-finger double domain	No	No	Orbicella faveolata	XP_020623631.1	1.00E-168	DNA binding
comp80166_c4_seq5	Fibrillin-1	C-type lectin-like domain, H-type lectin domain, Kazal type serine protease inhibitors and follistatin-like domains, TLD dc	No	Yes	Exaiptasia pallida	KXJ08513.1	0.00E+00	Microfibrils formation
comp80329_c2_seq2	Williams-Beuren syndrome chromosomal region 27 protein	Methyltransferase domain	No	No	Orbicella faveolata	XP_020620990.1	4.00E-70	Methyltransferase
comp73494_c0_seq1	Short-chain dehydrogenase/reductase family 9C member 7	Rossmann-fold NAD(P)H/NAD(P)(+) binding (NADB) domain	No	No	Nematostella vectensi	XP_001626221	9.00E-26	Retinol dehydrogenase activity
comp73218_c0_seq1	Putative diacyglycerol O-acyltransferase MT1468	Wax ester synthase-like Acyl-CoA acyltransferase domain	Yes	No	Acropora digitifera	XP_015771540.1	0.00E+00	Glycerol metabolic
comp63787_c0_seq1	Transmembrane protein-like	N/A						
comp73878_c0_seq1	Solute carrier family 25 member 36	Mitochondrial carrier protein	No	No	Stylophora pistillata	XP_022795900.1	5.00E-81	Pyrimidine transport
comp77373_c0_seq1	Prestin	SulP family	Yes (12)	No	Stylophora pistillata	XP_022800348.1	0.00E+00	Bicarbonate transport
comp66887_c0_seq1	Epidermal retinol dehydrogenase 2	NADB domain	No	No	Stylophora pistillata	XP_022788125.1	2.00E-171	Oxidoreductase
comp81225_c0_seq1	Putative gastrointestinal growth factor	Trefoil (P-type) domain	No	Yes	Orbicella faveolata	XP_020628961.1	2.00E-64	Uknown
comp33276_c0_seq2	Uncharacterized gene	N/A						
comp80659_c0_seq1	Hypothetical protein	Thyroid hormone-inducible hepatic protein Spot	No	No	Orbicella faveolata	XP_020612969.1	2.00E-102	Unknown
comp74456_c0_seq6	Uncharacterized gene	N/A						
comp71785_c0_seq1	Battenin	Nucleotide transporters	Yes (9)	No	Orbicella faveolata	XP_020610528.1	7.00E-108	Nucleotide transport
comp81878_c0_seq1	Cation transport regulator-like protein 1	GGCT-like domain	No	No	Orbicella faveolata	XP_020632724.1	6.00E-110	Inorganic ion transport and metabolism
comp53987_c0_seq1	Uncharacterized gene	N/A						
comp76481_c0_seq1	Protein phosphatase 1 regulatory subunit 3B	Carbohydrate binding domain	No	No	Orbicella faveolata	XP_020623779.1	3.00E-99	Glycogen metabolism
comp80594_c0_seq1	Lipid storage droplets surface-binding protein 2	Perilipin family	No	No	Orbicella faveolata	XP_020621197.1	1.00E-157	Lipid storage
comp77093_c2_seq3	Ammonium transporter Rh type C	Ammonium Transporter Family	Yes (12)	Yes	Stylophora pistillata	XP_022795556.1	0.00E+00	Ammonium transporter
comp82341_c0_seq1	F-box/LRR-repeat protein 7	F-box domain	No	No	Orbicella faveolata	XP_020608679.1	2.00E-153	Signal transduction regulation
comp92750_c0_seq1	Protein phosphatase 1 regulatory subunit 3D	Carbohydrate binding domain	No	No	Orbicella faveolata	XP_020631339.1	1.00E-43	Protein serine/threonine phosphatase
comp52813_c0_seq2	Uncharacterized gene	N/A						

Biology Open • Supplementary information
